# Supreme Court of the United States

**Published:** 1877-03

**Authors:** D. W. Middleton

**Affiliations:** C. S. C. U. S.


### Miscellaneous.

### SUPREME COURT OF THE UNITED STATES.

No. 113.—October Term, 1876.

| | |
|---|---|
| Daniel H. Smith, Appellant, *vs.* The Goodyear Dental Vulcanite Company and Josiah Bacon. | Appeal from the Circuit Court of the United States for the District of Massachusetts. |

Mr. Justice Strong delivered the opinion of the Court.

A brief review of the history and nature of the patent which the complainants allege has been infringed will aid materially in solving the questions presented by this appeal. On he 14th day of May, 1852, Dr. John A. Cummings, a dentist of Boston, filed in the Patent Office a caveat to protect an invention he claimed to have made, of certain new and useful improvements in the setting and plates of artificial sets of teeth. The description accompanying the caveat indicated with very considerable clearness what the alleged invention was, and the objects sought to be gained by it. The improvement was declared to "consist in forming the plate, and also the gums in which the teeth are inserted, of rubber or some other elastic substance, so compounded with sulphur, lead, and other similar substances as to form a hard gum, or whalebone gum, rigid enough for the purposes of mastication, and pliable enough to yield a little to the mouth." "By this improvement," the caveator said, "the teeth can be easily baked into the gums which form one piece with the plate." Subsequently on the twelfth of April, 1855, he applied for a patent, reciting in his application that he had previously entered a caveat. His accompanying specification declared the invention to consist in "forming the plate and gums to which the teeth are attached, of rubber or some other elastic material, so indurated as to be rigid enough for the purpose of mastication, and pliable enough to yield a little to the motions of the mouth, and in one piece, the teeth being imbedded in the elastic material while the material is in a soft condition, and then baked with the gums and plate, so that the teeth, gums, and plate will all be connected, forming, as it were, one piece." This application for a patent was rejected on the nineteenth of May next following, and the applicant was referred to two printed publications, one suggesting the use of gutta-percha as a base for artificial sets of teeth, and the other suggesting pastes, analogous to porcelain paste, as well as gutta-percha. Cummings then amended his specification by striking out all reference to gutta-percha or other merely elastic material, disclaiming the use of gutta-percha; and any material which is merely rendered plastic by heat and hardened by cooling, and he claimed the improvement in sets of mineral, or other artificial sets of teeth, which consists in combining the teeth with a rubber plate and gums, which, after the insertion of the teeth, are vulcanized by Goodyear's process, or any other process, forming thereby a cheap, durable, and elastic substitute for the gold plates theretofore used. This amendment, however, proved ineffectual. The application for a patent was again rejected, and a third rejection followed a reconsideration for which the applicant had asked. This third rejection was on the third day of February, 1856. From that time onward for several years, indeed until the patent was finally granted, the evidence very satisfactorily shows that Dr. Cummings was in a condition of extreme poverty, utterly unable to bear the necessary expenses of prosecuting his case further. But he did not withdraw his application. He did not ask for a return of part of the fee he had paid, nor by any act of his did he indicate acquiescence in the unfavorable action of the Patent Office. On the contrary, he continued to assert his expectation of ultimately obtaining a patent, formed plans for his own action after it should be obtained, and complained of what he supposed to be the negligence of his solicitor. The proof of his extreme poverty is ample. His ill-health interfered with his working successfully in the line of his profession, and his family was subjected to great privations. He seems never to have had any considerable money. He borrowed sometimes small sums to purchase underclothing for himself. He made frequent applications to his friends for advances to enable him to prosecute his application for a patent, offering as a compensation for such advances sometimes one-quarter and sometimes one-half of the patent when obtained. He appears never to have remitted his efforts until, in 1864, he induced Dr. Flagg, who had been his partner in former years, and Dr. Osgood to advance, first, one hundred dollars, and afterwards, seven hundred and twenty dollars, by means of which the patent was obtained. Even then he had not the twenty dollars necessary to be paid when it was allowed. For the assistance he thus received he gave one-quarter of his invention. Before this time, between the third rejection of his application and his obtaining the advances mentioned, everything was done which was within his power. In February, 1859, in the midst of his pecuniary embarrassments, his solicitor applied to the Patent Office, not for a return of any portion of the fee paid, nor for a withdrawal of the application, but that the specification and one drawing might be sent to him. This request was refused. An attempt was then made for an appeal to the board, but that not being allowed by the commissioner, nothing further was done in the Patent Office until the applicant was enabled by the funds obtained from Drs. Flagg and Osgood to renew his endeavors. Then, on the first of March, 1864, he presented a petition for the grant of a patent to himself for the same invention which he had endeavored to secure in 1855 (the application for which remained in the office unwithdrawn,) and accompanied his petition with a specification and drawings corresponding exactly with those he had previously made. This final effort was successful. The office practically acknowledged that the prior rejection had been an error, and declared that, in justice to his rights as an inventor, the admission of his claim, limited to the use of hard rubber or vulcanite, as he had before limited it, would not be objected to. Accordingly the patent was granted on the seventh of June, 1864, and by sundry conveyances it subsequently became vested in the complainants. Two surrenders and reissues have since been made, the last dated March 21, 1865, and it is for an alleged infringement of this second reissue that the present suit has been brought. The bearing of this history upon the merits of the controversy will appear as we proceed to examine the several defenses set up.

Among these the one perhaps most earnestly urged is the averment that the device described in the specification was not a patentable invention, but that it was a mere substitution of vulcanite for other materials which had previously been employed as a base for artificial sets of teeth, a change of one material for another in the formation of a product. If this is in truth all that the thing described and patented was; if the device was merely the employment of hard rubber for the same use, in substantially the same manner and with the same effect that other substances had been used for in the manufacture of the same articles, it may be conceded that it constituted no invention. So much is decided in Hotchkiss *vs.* Greenwood, 11 How., 248. But such is not our understanding of the device described and claimed. In the specification it is declared that the invention "consists in forming the plate to which the teeth, or teeth and gums, are attached, of hard rubber, or vulcanite, so called, an elastic material possessing and retaining in use sufficient rigidity for the purpose of mastication, and at the same time being pliable enough to yield a little to the motions of the mouth." This is immediately followed by a description of the manner of the proposed use; that is, of making the hard rubber plates, and the claim, as stated, is "the plate of hard rubber, or vulcanite, or its equivalent, for holding artificial teeth, or teeth and gums, substantially, as described," that is, plainly, formed as described. The invention, then, is a product of manufacture made in a defined manner. It is not a product alone separated from the process by which it is created. The claim refers in terms to the antecedent description, without which it can not be understood. The process detailed is thereby made as much a part of the invention as are the materials of which the product is composed. We shall not quote at large the description of the mode of making the plate. Such a quotation would unnecessarily prolong this opinion. It plainly shows a purpose of the inventor to secure what had not been secured before—a combination of a plate with artificial teeth, or with gums and teeth, in such a manner as to be free from the objections and defects or inconveniences attending the method before practiced of attaching such teeth to a metallic plate fitted to the roof of the mouth. Some of these objections are stated—such as expense, hurting the mouth, impeding mastication, and obstruction to perfect articulation. In carrying out the purpose proposed the materials employed were all old. The teeth, the wax, the plaster, the moulds, the soft rubber, and the hard rubber were none of them new. It is also true that the steps in the process were not all new. Plaster had been used for formation of moulds. The process of forming a plate by the use of such moulds was well known, and so was the process of converting a vulcanizable compound into vulcanite by heating it and allowing it to cool in moulds. But the process of Dr. Cummings extended beyond the use of known materials and the employment of the processes mentioned. It was vulcanizing soft rubber in a mould and in contact with artificial teeth inserted in place into it while it remained in a soft condition. It is well described by the circuit judge as "the making of a vulcanite dental plate out of a vulcanizable compound into which the teeth were imbedded in its plastic condition, and the rubber compound, with the teeth thus imbedded in it, afterwards vulcanized by heat, so that the teeth, gums, and plate should be perfectly joined without any intervening crevices, and the plate should possess the quality of hard rubber or vulcanite." The combination thus resulted in a manufacture which was "one piece." If, then, the claim be read, as it should be, in connection with the preceding part of the specification, and construed in the light of the explanation which that gives, the invention claimed and patented is "a set of artificial teeth as a new article of manufacture, consisting of a plate of hard rubber, with teeth, or teeth and gums, secured thereto in the manner described in the speeification, by imbedding the teeth and pins in a vulcanizable compound, so that it shall surround them while it is in a soft state, before it is vulcanized, and so that when it has been vulcanized the teeth are firmly and inseparably secured in the vulcanite, and a tight joint is effected between them, the whole constituting but one piece." It is evident this is much more than employing hard rubber to perform the functions that had been performed by other materials, such as gold, silver, tin, platinum, or gutta-percha. A new product was the result, differing from all that had preceded it, not merely in degree of usefulness and excellence, but differing in kind, having new uses and properties. It was capable of being perfectly fitted to the roof and alveolar processes of the mouth. It was easy for the wearer and favorable for perfect articulation. It was light and elastic, yet sufficiently strong and firm for the purposes of mastication. The teeth, gums, and plate constituting one piece only, there were no crevices between the teeth and their supports into which food could gather, and where it could become offensive, and there could be no such crevices so long as the articles lasted. They were unaffected by any chemical action of the fluids of the mouth. Besides all this, they were very inexpensive as compared with other arrangements of artificial teeth. To us it seems not too much to say that all these peculiarities are sufficient to warrant the conclusion that the device was different in kind or species from all other devices. We can not resist the conviction that devising and forming such a manufacture by such a process and of such materials was invention. More was needed for it than simply mechanical judgment and good taste. Were it not so, hard rubber would doubtless have been used in the construction of artificial sets of teeth, gums, and plates long before Cummings applied for his patent. To find a material, with a mode of using it, capable of being combined with the teeth, in such a manner as to be free from the admitted faults of all other known combinations, had been an object long and earnestly sought. It had been a subject for frequent discussion among dentists and in scientific journals. The properties of vulcanite were well known, but how to make use of them for artificial sets of teeth remained undiscovered and apparently undiscoverable until Cummings revealed the mode. But when revealed its value was soon recognized, and no one seems to have doubted the resulting manufacture was a new and most valuable invention. The eminent dentists and experts examined in this case uniformly speak of it as such. Not one has ventured to testify that it was not an invention. They speak of it as "a novel and desirable thing;" as "the greatest improvement in dentistry" made in many years, and as an invention which is "a great benefaction to mankind, whereby both health and comfort are promoted." The evidence also shows that it has wrought a revolution in dental practice, and that many thousands of operators are using it in preference to older devices. All this is sufficient, we think, to justify the inference that what Cummings accomplished was more than a substitution of one material for another—

more than the exercise of mechanical judgment and taste—that it was, in truth, invention. Undoubtedly the results or consequences of a process or manufacture may, in some cases, be regarded as of importance when the inquiry is whether the process or manufacture exhibits invention, thought, and ingenuity. Webster, on the subject-matter of patents, page thirty, says: "The utility of the change, as ascertained by its consequences, is the real practical test of the sufficiency of an invention, and since the one can not exist without the other, the existence of the one may be presumed on proof of the existence of the other. Where the utility is proved to exist in any degree, a sufficiency of invention to support the patent must be presumed." We do not say the single fact that a device has gone into general use, and has displaced other devices which had previously been employed for analogous uses, established in all cases that the latter device involves a patentable invention. It may, however, always be considered, and when the other facts in the case leave the question in doubt, it is sufficient to turn the scale.

We have, therefore, considered this branch of the case without particular reference to Hotchkiss *vs.* Greenwood, 11 How., 248. The patent in that case was for an improvement in making door and other knobs for doors, locks, and furniture, and the improvement consisted in making them of clay or porcelain, in the same manner in which knobs of iron, brass, wood, or glass had been previously made. Neither the clay knob nor the described method of attaching it to the shank was novel. The improvement, therefore, was nothing more than the substitution of one material for another in constructing an article. The clay or porcelain door knob had no properties or functions which other door knobs made of different materials had not. It was cheaper and perhaps more durable, but it could be applied to no new use, and it remedied no defects which existed in other knobs. Hence it was ruled that the alleged improvement was not a patentable invention. The case does decide that employing one known material in place of another is not invention, if the result be only greater cheapness and durability of the product. But this is all. It does not decide that no use of one material in lieu of another in the formation of manufacture can, in any case, amount to invention or be the subject of a patent. If such a substitution involves a new mode of construction, or develops new uses and properties of the article formed, it may amount to invention. The substitution may be something more than formal. It may require contrivance, in which case the mode of making it would be patentable, or the result may be the production of an analogous but substantially different manufacture. This was intimated very clearly in the case of Hicks *vs.* Kelsey, 18 Wall., 670, where it was said "the use of one material instead of another in constructing a known machine is, *in most cases*, so obviously a matter of mere mechanical judgment, and not of invention, that it can not be called an invention, unless some new and useful result, as increase of efficiency, or a decided saving in the operation be obtained." But where there is some such new and useful result, where a machine has acquired new functions and useful properties, it may be patentable as an invention, though the only change made in the machine has been supplanting one of its materials by another. This is true of all combinations, whether they be of materials or processes. In Crane *vs.* Price, 1 Webster's Patent Cases, 393, where the whole invention consisted in the substitution of anthracite for bituminous coal in combination with a hot-air blast for smelting iron ore, a patent for it was sustained. The doctrine asserted was that if the result of the substitution was a new, a better, or a cheaper article, the introduction of the substituted material into an old process was patentable as an invention. This case has been doubted, but it has not been overruled, and the doubts have arisen from the uncertainty whether any new result was obtained by the use of anthracite. In Kneass *vs.* The Schuylkill Bank, the use of steel plates instead of copper for engraving was held patentable. So has been the flame of gas instead of the flame of oil to finish cloth. These cases rest on the fact that a superior product has been the result of the substitution, a product that has new capabilities and that performs new functions. So in the present case the use, in the manner described, of hard rubber in lieu of the materials previously used for a plate, produced a manufacture long sought but never before obtained—a set of artificial teeth, light and elastic, easily adapted to the contour of the mouth, flexible, yet firm and strong, consisting of one piece, with no crevices between the teeth and the plate, impervious to the fluids of the mouth, unaffected by the chemical action to which artificial teeth and plates are subjected when in place, clean and healthy—peculiarities which distinguish it from everything that had preceded it. These differences, in our opinion, are too many and too great to be ascribed to mere mechanical skill. They may justly be regarded as the results of inventive effort, and as making the manufacture of which they are attributes a novel thing in kind, and consequently patentable as such.

A second objection urged by the defendant against the validity of the complainant's patent is alleged want of novelty of the invention, and a strenuous effort has been made to convince us that, although hard rubber had not been used in the manner described for the production of the manufacture, equivalent materials and processes had been, and that a plate substantially the same as that of Dr. Cummings had been made before his improvement. We are not, however, convinced. The patent itself is prima facie evidence that the patentee was the first inventor, at least it casts upon him who denies it the burden of sustaining his denial by proof. We do not find such proof in the case. Though the patent was not granted until June 7th, 1864, the invention was completed at least as early as April 12th, 1855, when the application for a patent was made. Indeed, as we have noticed, a caveat to protect it was filed on the 14th of May, 1852, which clearly foreshadowed the invention. Yet taking the spring of 1855 as the time when it was completed, we find nothing in the proofs to justify a conclusion that Dr. Cummings was not the first inventor. It would answer no good purpose to review the voluminous evidence supposed to bear upon this branch of the case. We shall refer only to that which is deemed most important, and which has been most pressed upon us in this argument. Of the English patent of Charles Goodyear it is enough to say that, though the provisional specification was filed March 14th, 1855, the completed specification was not until the 11th of September following. It was, therefore, on the last mentioned date that the invention was patented.

The experiments made by George E. Hawes, it must be admitted, closely resembled the process described in the reissued patent to the complainants. He cast in moulds sets of teeth on a tin base, in a manner very like that in which the vulcanite plate is formed by the Cummings process. But the experiments resulted in nothing practical. Hawes cast sets of teeth for the lower jaw only, the weight of the metal making the plate unfit for the upper. In consequence of the shrinkage of the metal in cooling, a tight joint could not be obtained between the teeth and the base. The sets were, therefore, liable to become offensive in consequence of deposits of food and the secretions of the mouth in the crevices. The shrinkage also prevented a close fitting of the plate to the roof of the mouth, and the tin base was affected by the chemical action of the secretions. In consequence of these and other objections the manufacture was soon abandoned, and it may properly be considered an abanoned experiment. It not only was not the same manufacture as that of Cummings, but it was not suggestive of it, and Dr. Hawes, who cast the tin plates, testifies that the use of vulcanite for dental purposes is the greatest improvement in his profession that he knew of in twenty-five years. He adds, "that vulcanite may be used by dentists in many ways which could not be accomplished by tin or platinum." In his opinion, therefore, the cast tin base was not substantially the same thing as the Cummings manufacture. So, also, Dr. Royce, who cast plates of tin for artificial teeth in a manner very similar to that of Dr. Hawes, testifies that the solid tin base was found practically unfit for the purpose, except in rare instances. He made but a few sets, none after 1850, and adopted the vulcanite, agreeing to pay for a license to use it in manufacturing dental plates.

We need go no farther into a consideration of the various devices and publications offered to show that the manufacture patented was known before Cummings invented it. Suffice it to say, that none of them, in our opinion, suggest or exhibit in substance such a manufacture. The defense of want of novelty is, therefore, not sustained.

It is further insisted by the defendant that the reissued patent on which this suit is founded is not a patent for the same invention which was described in the specification of the original patent, and, therefore, that the reissue is unauthorized and void. To sustain this position the defendant must overcome the presumption against him arising from the decision of the Commissioner of Patents in granting the issue, and this he can do only by showing from a comparison of the original specification with that of the reissue that the former does not substantially describe what is described and claimed in the latter. This must plainly appear before we can be justified in pronouncing the reissued patent void. But this, in our judgment, does not appear. The first specification describes a set of artificial teeth having a hard rubber plate made by a process substantially the same as that indicated in the latter patent. The description of the process is not quite so minute, but it is sufficiently full to be understood and to enable an operator to make the manufacture. Certainly it is not another process, and as its result is the same, it is impossible to hold that the reissued patent is for a different invention from the one protected by the original patent. It is true the specification of the reissue describes also another process not described in the specification of the first, namely, a mode of making the moulds, but that is not claimed as a part of the invention.

The remaining defenses to the bill rest mainly on the assumption that the new petition presented to the Patent Office in 1864 can not be regarded as a continuation of the application made for a patent on the twelfth of April, 1855. But this can not be conceded. The history of the application, as we have given it, forbids such an assumption. No act of Cummings amounted to a withdrawal of his first petition or to an acquiescence in its rejection. It is true he filed a second petition in 1864, but he accompanied it with substantially the specification that remained in the office and with the same drawings. It was a mode of procuring another consideration of his rejected claim, and the commissioner regarded it as such. The act of March 2d, 1861, gave him authority thus to regard it. He replied to the application that his claim was embraced in an application filed by him in 1855, and rejected for want of novelty, admitted that it had been improperly rejected, and suggested an amendment to make it correspond with his former amended claim. It is impossible, in view of these facts, to regard the effort to obtain a new patent in 1864 as a new and independent application, disconnected from the application made in 1855. It was but one stage in a continuous effort. In Godfrey *vs.* Eames, 1 Wall., 317, the first application was actually withdrawn and a new petition was presented at the time of the withdrawal, with a different description of the invention, but as the thing patented under the second might have been engrafted as an amendment of the first, it was ruled that all the proceedings constituted one application. This court said, "If a party choose to withdraw his application for a patent, and pay the forfeit, intending at the time of such withdrawal to file a new petition, and he accordingly does so, the two petitions are to be considered parts of the same transaction, and both as constituting one continuous application." We are not aware that filing a second petition for a patent, after the first has been rejected, has ever been regarded as severing the second application from the first and depriving the applicant of any advantage he would have enjoyed had the patent been granted without a renewal of the application. The contrary was decided by the Circuit Court for the Southern District of Ohio, in Bell *vs.* Daniels, 1 Fisher, 372, and in Blandy *vs.* Griffith et al., 3 Fisher, 609. And these decisions are founded in justice and sound reason.

If, then, as we think it must be held, the proceeding to obtain the patent was a continuous one from 1855 until it was granted; if the application of 1855 is not severable from the proceedings of 1864, there is no foundation whatever for the allegation that the invention was abandoned to the public, and that it was in public use or on sale for more than two years before the inventor's application. The first use of it proved, by any other than Dr. Cummings, was in 1859, and there is no evidence that this was with his consent. And the proof respecting his health and pecuniary condition, together with his constant efforts to obtain the necessary means to prosecute his right, rebuts all presumption that he ever abandoned, actually or constructively, either his invention or his application for a patent. That he never intended an abandonment of his invention is perfectly clear, and it was not his fault that granting the patent was so long delayed.

The conclusion of the whole matter is that the patent is a valid one, and, therefore, that the decree of the circuit court should be affirmed.

The decree of the circuit court is affirmed.

---

Mr. Justice BRADLEY dissenting.

I dissent from the judgment of the court in this case on the ground that the patentee having duly made his application for a patent in 1855, and the same having been three times rejected, must be considered as having abandoned the same, inasmuch as no further effort was made to obtain a patent until eight years afterwards, without any pretense that the patentee was engaged in perfecting his invention, and in the meantime the invention which he claims as his had come into general public use. The application for a patent made in 1864 was a new and independent application, and should be treated as such. As the public had enjoyed the use of the invention for more than two years prior to this application, the patent should be declared invalid. Great injustice will, in my judgment, be done to the public to allow a patent obtained under such circumstances to stand. The public had a right to suppose that no further application would be made. The levy of a tribute now on all the dentists of the country who have brought the plate into public notice and use seems to me a species of injustice. The delay of the patentee, in fact, is made to operate to his benefit instead of his prejudice; his patent being made to run eight years longer than it would have done had it been granted when first applied for. So that the public is still further injured by sustaining the patent as finally granted. It is too common a case that associated companies, in order to maintain some valuable monopoly, look about to see what abandoned invention or rejected application, or ineffective patent, can be picked up, revamped and carried through the Patent Office; and by the aid of ingenious experts and skillful counsel succeeded in getting the. desired protection. I think that the courts ought to be strict in maintaining the rights of the public in such cases. And the present case seems to me to be one in which we ought to hold the patent invalid as against those rights.

Mr. Justice Miller and Mr. Justice Field concur in this opinion.

D. W. Middleton,

*C. S. C. U. S.*

—*Cosmos.*

